# Human genomics of COVID-19 pneumonia: contributions of rare and common variants

**DOI:** 10.1146/annurev-biodatasci-020222-021705

**Published:** 2023-05-17

**Authors:** Aurélie Cobat, Qian Zhang, COVID human genetic effort, Laurent Abel, Jean-Laurent Casanova, Jacques Fellay

**Affiliations:** 1 Laboratory of Human Genetics of Infectious Diseases, Necker Branch, INSERM U1163, Paris, France; 2 University Paris Cité, Imagine Institute, Paris, France; 3 St. Giles Laboratory of Human Genetics of Infectious Diseases, Rockefeller Branch, The Rockefeller University, New York, NY, USA; 4 Howard Hughes Medical Institute, New York, NY, USA; 5 Department of Pediatrics, Necker Hospital for Sick Children, Paris, France; 6 School of Life Sciences, École Polytechnique Fédérale de Lausanne, Lausanne, Switzerland; 7 Swiss Institute of Bioinformatics, Lausanne, Switzerland; 8 Precision Medicine Unit, Biomedical Data Science Center, Lausanne University Hospital and University of Lausanne, Lausanne, Switzerland

**Keywords:** SARS-CoV-2, COVID-19 pneumonia, GWAS, Inborn errors of immunity, Type I interferons

## Abstract

SARS-CoV-2 infection is silent or benign in most infected individuals, but causes hypoxemic COVID-19 pneumonia in about 10% of cases. We review here studies of the human genetics of life-threatening COVID-19 pneumonia, focusing on both rare and common variants. Large-scale genome-wide association studies have identified more than 20 common loci robustly associated with COVID-19 pneumonia with modest effect sizes, some implicating genes expressed in the lungs or leukocytes. The most robust association, on chromosome 3, concerns a haplotype inherited from Neanderthals. Sequencing studies focusing on rare variants with a strong effect have been particularly successful, identifying inborn errors of type I IFN immunity in 1–5% of unvaccinated patients with critical pneumonia, and their autoimmune phenocopy, autoantibodies against type I IFN, in another 15–20% of cases. Our growing understanding of the impact of human genetic variation on immunity to SARS-CoV-2 is enabling health systems to improve protection for individuals and populations.

## Introduction

More than 600 million cases of severe acute respiratory syndrome coronavirus 2 (SARS-CoV-2) infection and at least 6.5 million deaths from COVID-19 have already been recorded worldwide (1). The clinical manifestations of COVID-19 are highly variable, ranging from silent infection to life-threatening disease, typically beginning with pneumonia. Before effective anti-SARS-CoV-2 vaccines became available, ~ 3% of infected individuals developed critical COVID-19 pneumonia requiring supplementary high-flow oxygen (O_2_> 6 L/min), mechanical ventilation (non-invasive or by intubation), or extracorporeal membrane oxygenation (ECMO) (2), with an estimated infection fatality rate of about 0.5–1% (3, 4). Advanced age was, by far, the strongest predictor of COVID-19 severity at the time, with the risk of death doubling with every five years of age from childhood onward (3, 4). Unvaccinated men also have a 1.5 times greater risk of death than women (3, 5, 6). Ancestry, social status, and several comorbid conditions have been associated with higher disease severity and death rates, but with modest odds ratios (OR, typically <1.5, rarely >2) (5, 7–9). In the early days of the pandemic, it was already obvious that demographic and clinical factors did not entirely account for the marked inter-individual variability of COVID-19 clinical manifestations. The hypothesis of human genetic predisposition was most strongly supported by the rare cases of previously healthy young individuals being admitted to intensive care for respiratory failure. Other clinical presentations have emerged during the pandemic, including multisystem inflammatory syndrome (MIS) in children (MIS-C) (10) and adults (MIS-A) (11), “COVID-toes” (pernio) (12), and long-term neurocognitive, pulmonary, and musculoskeletal sequelae collectively referred to as “long COVID” or “post-acute COVID-19 syndrome” (13). Here again, clinicians were puzzled by the remarkable differences in clinical manifestations observed at population level and suggestive of a causal or modulating role for human genetic variation (2).

The development of anti-SARS-CoV-2 vaccines rapidly became a global health priority. The massive deployment of several effective vaccines, developed in less than a year, has indubitably altered the course of the pandemic, largely decreasing the risks of severe disease, hospitalization, and death in regions of high vaccine coverage (14). However, the success of vaccination has been jeopardized by the limited access to vaccination in lower-income countries, vaccine hesitancy, and the emergence of multiple variants of concern, such as Alpha (B1.1.7), Beta (B.1.351), Delta (B.1.617.2) and Omicron (BA.1, BA.2, BA.4, BA.5 and BQ.1), all of which are more transmissible than the original strain, and some of which increase the risk of severe disease (15–19) and/or immune escape (20). Moreover, even the most effective RNA vaccines do not prevent infection *per se*, sometimes resulting in “breakthrough” pneumonia in vaccinated individuals (21, 22). Hence, the fight against this disease continues, and humanity will benefit greatly from improvements in our understanding of the mechanisms of host defense and immune protection against SARS-CoV-2. Human genetic studies of infectious diseases can point to the primary cause of disease and provide invaluable mechanistic insights (23, 24). In this review, we will briefly introduce the field of human genetics of infectious diseases, and will describe the main genetic findings relating to susceptibility to COVID-19 pneumonia and its severity, with their downstream implications.

## 1. Human genetics of infectious diseases

For almost all infectious agents, the clinical manifestations of infection are highly variable, ranging from silent infection to lethal disease (25). The field of human genetics of infectious diseases aims to characterize the genetic variants accounting for this considerable interindividual variability. It was long held as a dominant paradigm that rare infections with weakly virulent microbes and/or multiple, recurrent infections in a single patient result from rare monogenic inborn errors of immunity (IEIs, also called primary immunodeficiencies [PIDs]), whereas common infections with more virulent pathogens are more influenced by the polygenic inheritance of common alleles (26). IEIs were discovered through individual-based studies focusing on a small number of patients, sometimes even a single patient, initially with sporadic infections. IEIs may be individually rare, but, collectively, they are more common than initially thought and their study can unravel general mechanisms of diseases that can be triggered by other causes (23, 24).

At population level, attempts to understand how human genetic variation modulates the individual response to more common pathogens began seven decades ago. One of the first major discoveries was the identification of multiple red blood cell abnormalities associated with a lower risk of severe malaria and subject to strong positive selection in populations living in regions in which malaria was endemic (27). However, host genetic studies with the objective of discovering variants were long hampered by technological limitations: the low throughput of DNA analysis methods at the time obliged researchers to use candidate gene approaches, unless they had access to family samples, which allowed more solid linkage studies. Most candidate gene studies had relatively small sample sizes and failed to account for population stratification or multiple testing, generating a flurry of false-positive results. Recent progress in large-scale genotyping and sequencing technology, coupled with dramatic improvements in bioinformatics and data science, have finally made it feasible to mine the full human genome for infectious disease-altering variants. Genome-wide association studies (GWAS) and deep sequencing analyses of individuals with unusually severe clinical presentations have both been used successfully, and in a highly complementary fashion, to explore the genetic architecture of human susceptibility to infections. GWAS, mostly based on genome-wide genotyping arrays, have identified many associations between common human genetic variants — generally defined as variants with a minor allele frequency of at least 1% — and complex traits or diseases. They have made it possible to identify chromosomal loci associated with the natural course of disease and responses to treatment in populations infected with HIV-1, hepatitis B and C viruses, *Plasmodium falciparum*, *Mycobacterium tuberculosis* and *M. leprae*, for example (25). However, the risk factors identified were no more than modest at individual level.

The recent development of next-generation sequencing technologies, making it possible to identify rare coding variants rapidly at genome-wide scale through whole-exome (WES) or whole-genome sequencing (WGS), has revolutionized the field of human genetics. It has accelerated the discovery of new disease-causing genes and provided molecular insight into the etiology of many IEIs (28, 29). Over the last two decades, we and others have demonstrated that rare monogenic IEIs underlie a growing number of life-threatening viral, bacterial, fungal, and parasitic infections in otherwise healthy individuals with normal resistance to other infectious agents (23, 24, 30, 31). Most of these IEIs are not Mendelian, as they frequently display incomplete penetrance. In addition, they are genetically heterogeneous, with both locus and allelic heterogeneity, with different mutations of several genes underlying the same infectious phenotype, but often united by the same signaling pathway (i.e. physiological homogeneity) (30). For example, Mendelian susceptibility to mycobacterial disease (MSMD) is caused by inborn errors of IFN-γ immunity (32–34), with mutations of 15 genes and 30 allelic forms already reported. Likewise, forebrain herpes simplex encephalitis (HSE) can be caused by IEI of TLR3-dependent type I IFN immunity resulting from mutations of eight genes (35, 36). Since 2001, rare monogenic defects have also been shown to underlie some common infectious diseases in rare patients, as exemplified by the identification of several patients with tuberculosis but no familial history of clinical MSMD carrying causal mutations of MSMD genes (37). These rare IEIs led to the recent discovery of a more common IEI, homozygosity for the P1104A TYK2 allele, underlying about 1% of cases of tuberculosis in populations of European descent (38–40).

Building on these achievements and long-standing international collaborations in human genomics dating back to the Human Genome Project, the HapMap project and the 1000 Genomes Project, the host genetic research community quickly came together in the early weeks of the COVID-19 pandemic, to search the human genome for potential clues relating to viral pathogenesis and host immune responses. Several consortia were created, including the COVID-19 Host Genetic Initiative (HGI, https://www.covid19hg.org) (41) and the Covid Human Genetic Effort (Covid-HGE, https://www.covidhge.com) (42). Existing resources were enhanced and redirected to facilitate the rapid analysis of large numbers of patients and controls. A prime example of this is provided by the GenOMICC study (Genetics Of Mortality In Critical Care, https://genomicc.org), which had been recruiting patients in the United Kingdom since 2015 for the study of emerging infections, sepsis and other forms of life-threatening illness. This study collected clinical data samples from thousands of critically ill COVID-19 patients and generated full genome sequence data in record time. The global effort was not restricted to the academic sector either: several direct-to-consumer companies used their large reservoir of clients (who agreed to be recontacted for research purposes) to perform genome-wide analyses of susceptibility to infection and symptom severity.

## 2. Human genetics of COVID-19 pneumonia

Numerous human genetic studies have investigated the genetic determinants of COVID-19 pneumonia. As discussed in the corresponding sections, large-scale GWAS approaches, mostly based on genome-wide SNP arrays and single-variant statistics assuming an additive genetic model, led to the identification of multiple common genetic variants associated with a modest increase in the risk of COVID-19 pneumonia. High-throughput DNA sequencing approaches, such as WES or WGS for the identification of rare genetic variants, have also been highly successful, resulting in the discovery of genes and pathways crucial for the control of SARS-CoV-2 infection through rare-variant aggregation tests followed by functional validation.

### a. Common variants

**Figure 1.**
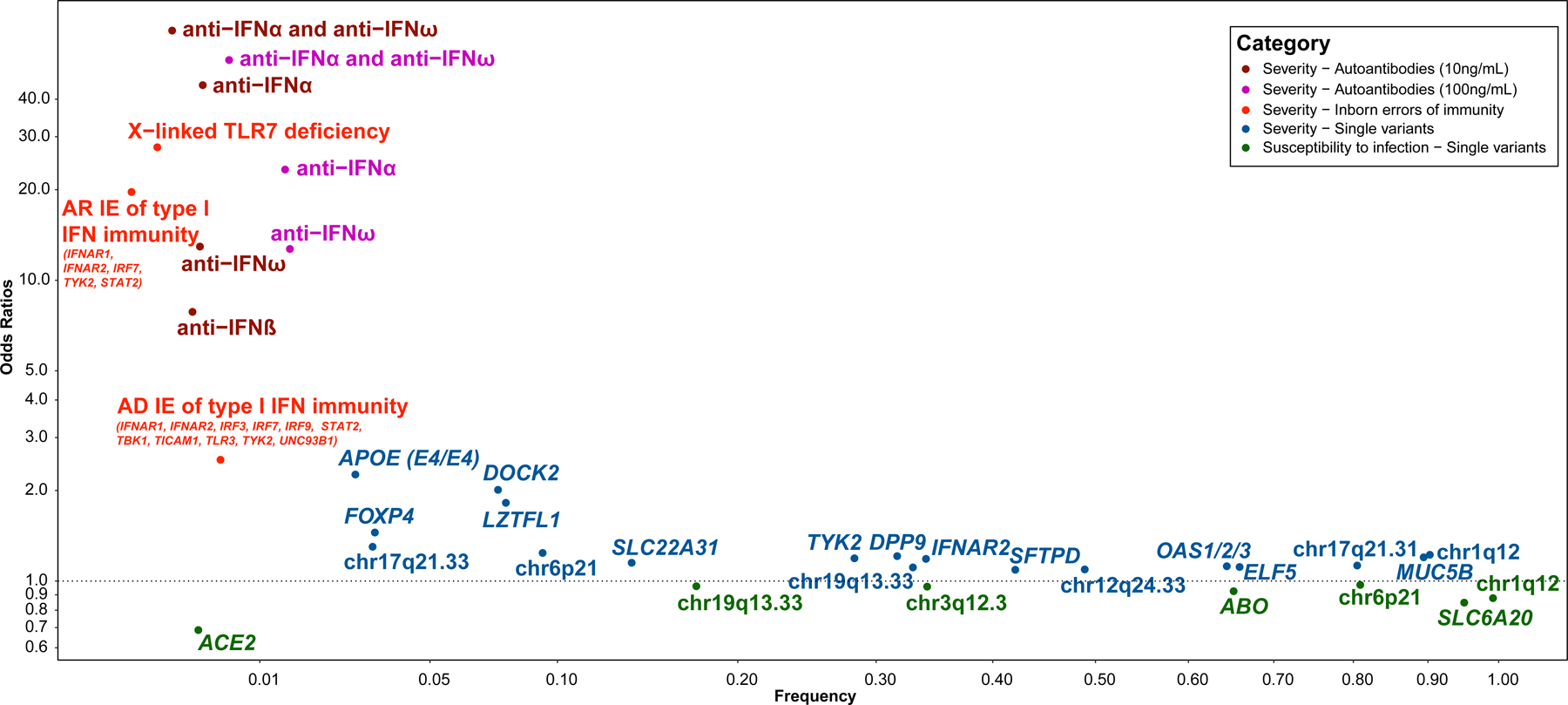
Genetic and immunological determinants of COVID-19 pneumonia. Odds ratios (OR) for the associations of auto-Abs against type I IFN, inborn errors of immunity (IEIs) and single genetic variants with the severity of COVID-19 pneumonia and resistance to SARS-CoV-2 infection are plotted according to risk factor frequency. For auto-Abs against type I IFN, the ORs and frequency were taken from (114). For X-linked TLR7 deficiency, the OR for the aggregated effect of biochemically proven loss of function (LOF) was taken from (74) and the cumulative frequency of biochemically proven LOF was taken from (76). For other IEIs, the OR for the aggregated effect of homozygous (autosomal recessive, AR) or heterozygous (autosomal dominant, AD) predicted LOF was taken from (74) and the corresponding cumulative frequencies were estimated from Gnomad v2.1.1. For single variants, the OR, assuming an additive model, and deleterious allele frequencies were taken from the most recent update of the COVID-19 Host Genetics Initiative GWAS study (43), except for the *DOCK2* and *APOE* loci. The chromosomal region or closest gene is indicated. For the *DOCK2* locus, the OR for the effect of the rs60200309-A variant on the severity of COVID-19 pneumonia under an additive model and allele frequency for rs60200309-A were taken from (156). For the *APOE* locus, the hazard ratio for the effect of APOE4 homozygosity as opposed to APOE3 homozygosity for COVID-19 mortality and the frequency of APOE4 homozygosity were taken from (157).

Multiple large-scale GWAS have investigated the genetic factors associated with disease severity by comparing COVID-19 pneumonia patients stratified into various subgroups (hospitalized, requiring ventilation, admitted to the ICU, or deceased) with SARS-CoV-2 infected individuals with asymptomatic or mild clinical presentations, or untested individuals from the general population. More than 15 genomic regions harboring common variants (Figure 1) have already been robustly associated with COVID-19 pneumonia (43, 44). The strongest signal — and the very first to be reported, in the spring of 2020 — is that for the 3p21.31 locus, where a Neanderthal haplotype was found to be associated with an OR of 1.8 for severe infection (43, 45–48). The association with this locus has been replicated in several independent cohorts. The frequency of the lead SNP ranges from 1% in East Asians to 9% in Europeans and 23% in South Asians, but there is no evidence of between-origin heterogeneity (43). It has proved challenging to identify the causal genes and variants underlying this association, due to long-range linkage disequilibrium in the region and a high local density of genes with a known or putative role in immunity. *In silico* functional analyses identified *LZTFL1* as the most probable candidate gene (49), but variants of the chemokine receptor genes *CCR9*, *CXCR6* and *XCR1* may also contribute to the association signal. Some of the additional loci implicated point to a role for known immune-related genes or genes known to be involved in lung function, as detailed below. Surprisingly, given the very high level of polymorphism of both class I and class II HLA genes and their demonstrated role in modulating multiple infectious diseases, the few associations identified in the HLA region have proved to be weak. It was not until very large meta-analyses were performed relatively late in the course of the pandemic that genome-wide significant association signals were confirmed for polymorphisms of both class I and class II HLA genes (43, 47, 48).

The loci pointing to a role for known immune-related genes include the *IFNAR2* gene, encoding the interferon (IFN) alpha/beta receptor 2, for which associations with variants have been replicated in several studies (47, 50). Type I IFNs play a central role in the innate immune response to SARS-CoV-2, as demonstrated by the marked increase in the risk of life-threatening disease associated with rare loss-of-function variants of interferon-related genes and anti-IFN autoantibodies, as discussed below. It is, therefore, unsurprising that common variants of a subunit of the type I IFN receptor modulate the severity of infection, particularly as the top-ranking associated variants are expression quantitative trait loci (eQTL) for *IFNRA2*. An association with a *TYK2* variant has been identified, which is particularly interesting, given the key role of TYK2 in infection and immunity. The common rs34536443 (p.Pro1104Ala) variant, which is protective against some autoimmune diseases (51) but increases the risk of tuberculosis in homozygous individuals (40), has been shown to be associated with a modest increase in the risk of severe COVID-19 (48). Rare loss-of-function *TYK2* variants are discussed below. GWAS have also identified a common haplotype of Neanderthal origin encompassing the *OAS1/2/3* genes on chromosome 12 (12q24.13) that is associated with the risk of hospitalization for COVID-19 (47, 48, 52, 53). The OAS proteins are cytosolic type I IFN-inducible antiviral proteins (54, 55). One of the candidate causal variants in this region is the *OAS1* splice variant rs10774671. The minor and reference G allele at rs10774671, which provides weak protection against severe forms of COVID-19, encodes a longer and more active form of OAS1 (56).

In their most recent meta-analysis including more than 150,000 cases (43), the COVID-19 HGI reported multiple associations between disease severity and genes involved in normal lung function. First, a regulatory variant (rs35705950:G>T) mapping to the promoter region of *MUC5B* was found to be associated with a lower risk of hospitalization. The minor T allele is associated with higher levels of *MUC5B* mRNA and a higher risk of idiopathic pulmonary fibrosis (57). Second, a missense variant of *SFTPD* (rs721917:A>G, p.Met31Thr) was found to be associated with more severe respiratory symptoms. *SFTPD* encodes the surfactant protein D, and the same alternative allele was previously shown to have a negative impact on lung function (58) and to increase the risk of chronic obstructive pulmonary disease (59). Third, a missense variant of *SLC22A31* (rs117169628:G>A, p.Pro256Leu) was also found to be associated with a higher risk of hospitalization. *SLC22A31* encodes a solute carrier protein that is highly expressed in the lung. Finally, a strong association with severe disease was observed with an intronic variant of *DPP9* (rs2109069:G>A) previously reported to increase the risk of idiopathic pulmonary fibrosis (60). These variants have very small effect sizes (with odds ratios between 0.8 and 1.2), but their study may improve our understanding of SARS-CoV-2 pathogenicity by shedding light on the underlying molecular mechanisms.

### b. Rare variants

#### Type I IFN influenza susceptibility loci

Based on our discoveries over the last 20 years, we launched the Covid-HGE, an international consortium, with the aim of deciphering the human genetic and immunological basis of the various clinical manifestations of SARS-CoV-2 infection. The first breakthrough emerged from a study testing the hypothesis that candidate inborn errors of TLR3-, IRF7-, and IRF9-dependent type I IFN immunity previously shown to underlie life-threatening influenza pneumonia (2, 23, 24, 30, 31, 61–64) might also underlie critical COVID-19. We considered the three loci (*IRF7*, *IRF9* and *TLR3*) for which germline mutations are causal for influenza pneumonia (62–64) and 10 other genes (*IFNAR1, IFNAR2, IRF3, IKBKG, STAT1, STAT2, TBK1, TICAM1, TRAF3* and *UNC93B1*) encoding products biochemically and immunologically connected to the three core genes, for which deleterious genotypes have been shown to underlie other severe viral diseases (61). We screened a cohort of 659 patients with critical COVID-19 for rare variants predicted to be loss-of-function (pLOF) at these 13 type I IFN influenza susceptibility loci. We found a significant enrichment in these variants in patients with critical COVID-19 relative to 534 SARS-CoV-2-infected controls who remained asymptomatic or paucisymptomatic, with mild, self-healing, ambulatory disease (*P* = 0.01) (61). We also found that 23 (3.5%) of the patients with critical COVID-19 carried biochemically deleterious germline mutations of eight of the 13 genes. These patients included four unrelated previously healthy adults aged 25–50 years with autosomal recessive (AR) complete IRF7 or IFNAR1 deficiency. AR IFNAR1, IFNAR2, TBK1, and STAT2 deficiencies were subsequently reported in children with critical COVID-19, and AR TYK2 deficiency was identified in children with COVID-19 pneumonia (65–69).

**Figure 2.**
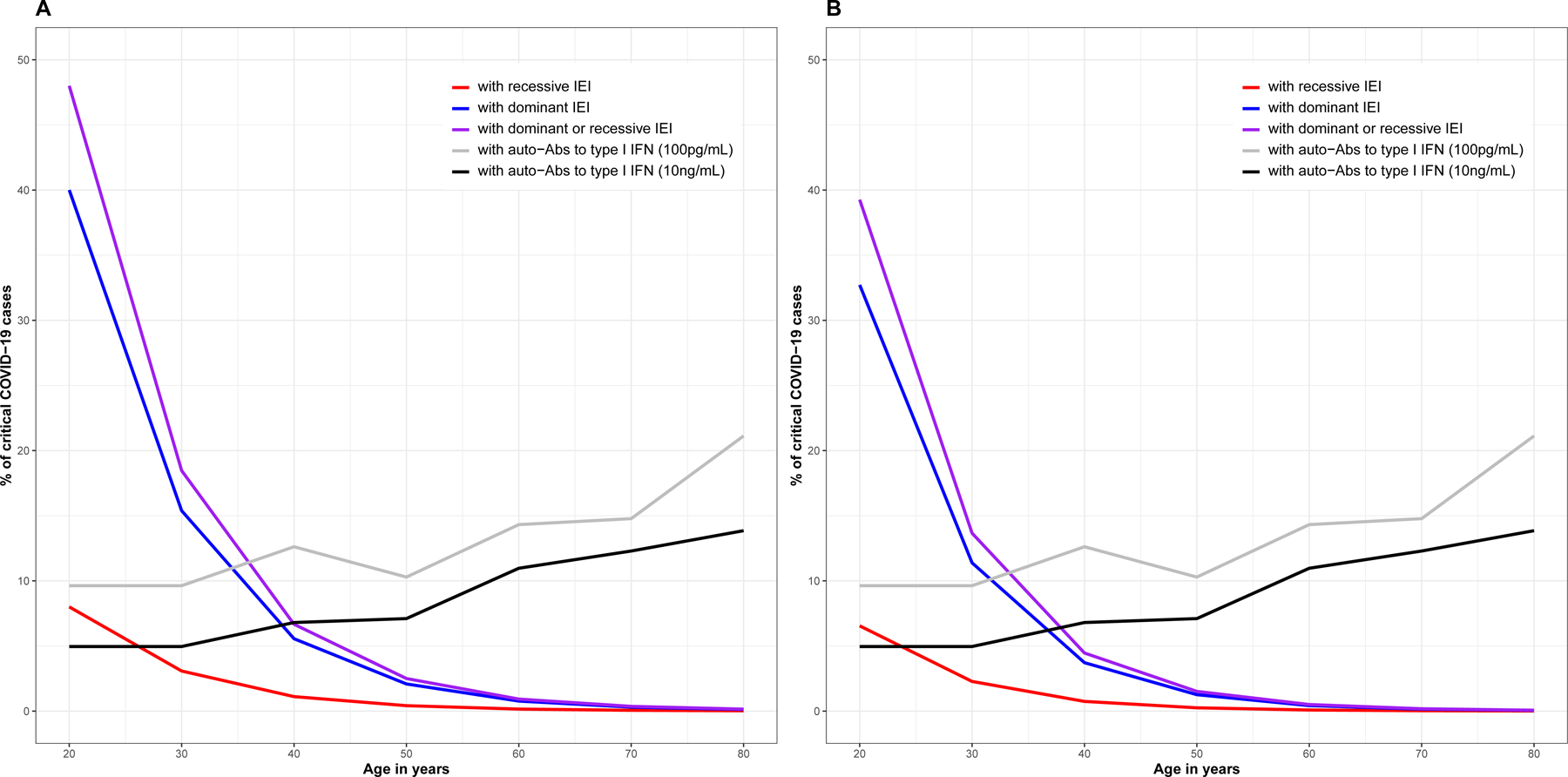
Proportion of critical COVID-19 patients with IEIs and auto-Abs against type I IFN as a function of age. Age-specific proportions of dominant and recessive IEIs among cases of critical COVID-19 pneumonia were estimated with Bayes theorem as a function of the probability of critical COVID-19 pneumonia for IEI carriers infected with SARS-CoV-2 (i.e. the penetrance). The age-specific frequency of IEIs in the general population and the age-specific critical infection rate were taken from (158). The age-specific frequency of IEIs in the general population was estimated from the frequency of IEIs at birth, assuming a non-specific mortality rate (i.e. not attributable to COVID-19) of 0 (panel A) or 1% (panel B) per year. Based on our previous findings (67, 159, 160), the frequency of IEIs at birth was set at 10^−3^ for dominant and 5 × 10^−4^ for recessive IEIs. We assumed a penetrance of 0.2 for dominant IEIs and 0.8 for recessive IEIs, consistent with the larger effect size estimated for recessive than for dominant IEIs. The age-specific proportions of patients with critical COVID-19 producing auto-Abs neutralizing low doses (100 pg/mL; gray line) or high doses (10 ng/mL; black line) of IFN-α and/or IFN-ω were taken from (114).

Several other groups were unable to replicate our findings (44, 70–72). There are several possible reasons for this (73), two of which are particularly important. First, the key epidemiological factor driving COVID-19 severity was ignored: our international cohort was much younger than the other cohorts (mean age of 52 vs. 66 years). As discussed below, these inborn errors are much more frequent in patients under the age of 60 years (Figure 2). Second, the other groups did not test for auto-Abs against type I IFN, the most common determinant of critical COVID-19, especially in patients over 60 years old (discussed in a specific section below). More recently, we confirmed an enrichment in rare pLOF variants at the 13 type I IFN-related influenza susceptibility loci in an extended sample of 3269 patients with critical COVID-19 relative to 1373 controls with asymptomatic or mild infection (*P* = 2.1×10^−4^) (74). The addition of *TYK2* strengthened the association signal, particularly if a recessive model was assumed. We also found that homozygous carriers of rare pLOF variants had a higher risk of life-threatening COVID-19 than heterozygotes, consistent with the expected higher penetrance of recessive IEIs than of dominant IEIs. In analyses restricted to rare in-frame nonsynonymous variants, we detected no significant enrichment in patients relative to controls. This result was not surprising, as we showed in a previous study (61) that less than 15% of the rare in-frame nonsynonymous variants at the 13 loci were biochemically proven LOF variants (bLOF), whereas all the pLOF variants were found to be bLOF variants. A similar trend was observed for other immune system genes, even when advanced *in silico* scoring systems, such as CADD score, were used to stratify the variants (39, 75–77). The study of in-frame nonsynonymous variants will therefore require the experimental characterization of all these variants.

#### Unbiased screening for chromosome- and genome-wide rare variant burden

**Figure 3.**
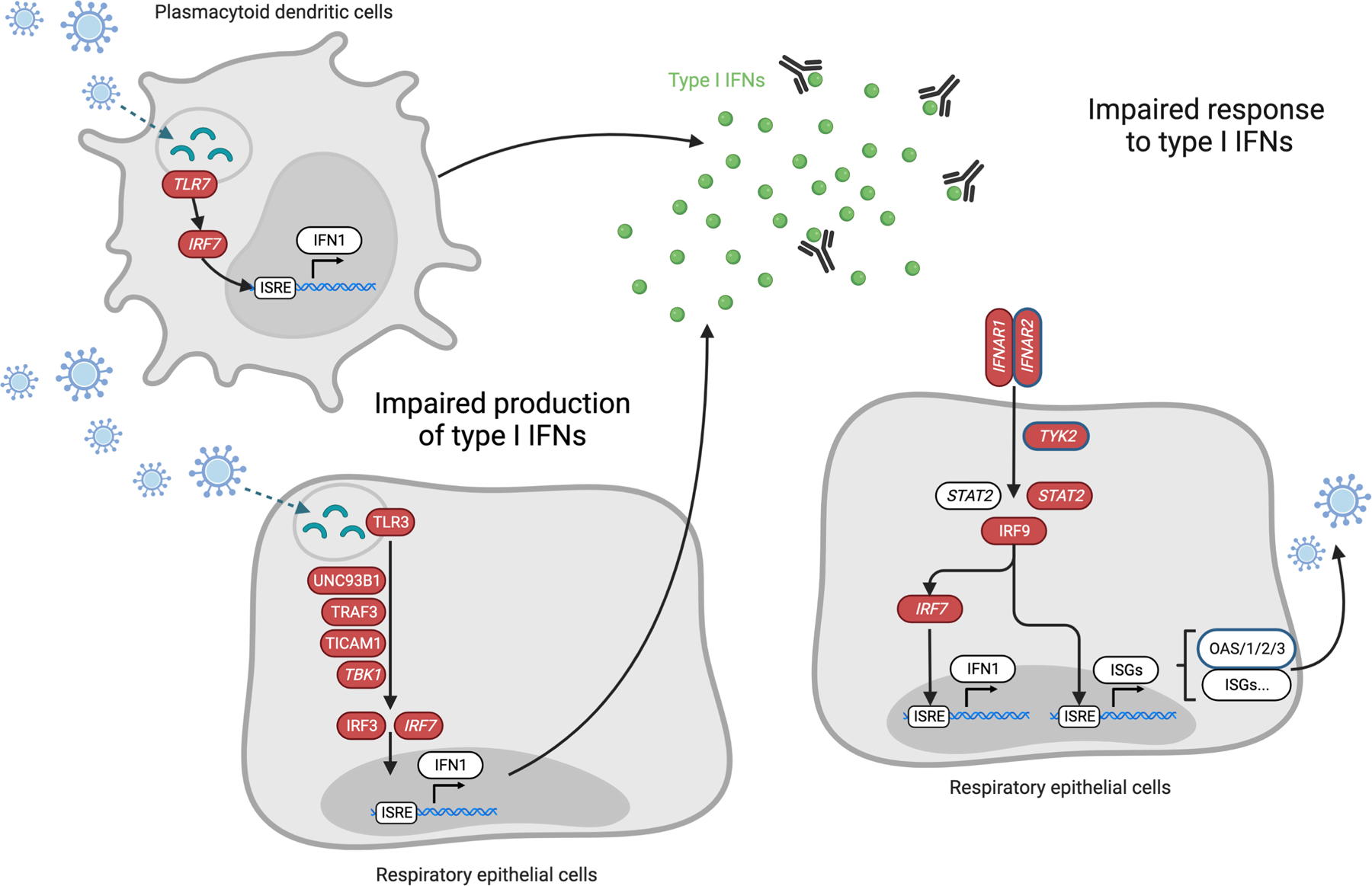
Impaired type I IFN immunity underlies life-threatening COVID-19 Nearly 20% of patients with life-threatening COVID-19 pneumonia have impaired production of or response to type I interferons (IFNs), due to inborn errors of immunity (IEIs, 1 to 5%) or to blockade of type I IFN activity by pre-existing neutralizing autoantibodies (~15%). IEI of type I IFN immunity (genes shown in red) have been identified and lead to: (1) an impaired production of type I IFNs (left) in respiratory epithelial cells (RECs) and/or in blood plasmacytoid dendritic cells (pDCs), or (2) an impaired response to type I IFNs (right) in RECs, in response to SARS-CoV-2 infection. Genes for which X-linked or autosomal recessive defects have been identified are in italic. Genes for which common variants have been associated by GWAS with severe COVID-19 are circled in blue.

We hypothesized that the higher risk of critical COVID-19 in men than in women might be explained by X-linked disorders. We conducted an unbiased X chromosome-wide gene burden test on a cohort of 1,202 unrelated, autoantibody-negative, male patients with critical COVID-19 pneumonia and 331 men with asymptomatic or mild COVID-19. The most strongly associated gene, and the only gene remaining significant after accounting for the number of genes tested, was *TLR7*, with 21 unrelated patients carrying a very rare hemizygous nonsynonymous variant that was completely absent from controls (*P* = 3.5 × 10^−5^) (76). Sixteen of these 21 patients carried a *TLR7* allele that was loss-of-function or hypomorphic. Biochemically proven TLR7 deficiency was identified in four additional male patients with critical COVID-19 (74), and four male patients with severe COVID-19 (67, 76) from the Covid-HGE cohort. Moreover, we confirmed the proposed diagnosis of TLR7 deficiency in nine of other 16 reported male patients (65, 78–82), with our biochemical assay (76). An enrichment in rare nonsynonymous *TLR7* variants in patients with critical COVID-19 was reported in another two studies, but the variants were not disclosed and the diagnosis of TLR7 deficiency remains to be confirmed, especially in the women (70, 71). Overall, TLR7 deficiency was found to account for about 1% of cases of critical COVID-19 in men (74, 76). The penetrance of TLR7 deficiency for severe or critical COVID-19 among relatives of index cases was high, but incomplete, especially in children. Human TLR7 is an endosomal receptor of ribonucleic acids expressed by B cells and myeloid subsets. Its stimulation in plasmacytoid dendritic cells (pDCs) results in the production of large amounts of type I IFN (76). We showed that blood B-cell lines and myeloid cell subsets from patients with TLR7 deficiency did not respond to TLR7 stimulation and that the patients’ pDCs produced low levels of type I IFNs in response to SARS-CoV-2, further highlighting the essential role of type I IFN for protection against SARS-CoV-2 (Figure 3) (76).

Several large-scale sequencing studies have attempted to identify new genetic causes of severe COVID-19 pneumonia through unbiased genome-wide gene burden analyses (44, 70, 71, 74). None of the genes considered remained statistically significant after stringent correction for the number of genes and phenotypes tested. In addition to *TLR7*, for which association has consistently been reported across studies (70, 71, 74, 76, 78), two genes reached the less conservative exome-wide significance threshold of 2.5×10^−6^ in one study focusing on 5,085 individuals with critical COVID-19 and 571,737 controls, mostly uninfected, from the general population (70): *MARK1* (*P* = 1.9×10^−6^) and *RILPL1* (P = 2.4×10^−6^), with cases displaying an enrichment in pLOF variants. Nevertheless, these results require further investigation. It should be stressed that stringent correction for multiple testing, while necessary to avoid false positives, is a conservative strategy, and that a lack of formal statistical significance at genome-wide level does not exclude biological causality and medical significance. The burden of proof can be provided experimentally via biochemical, virological, and immunological experiments, as we previously did for TLR7 (76). Additional genes may be found by restricting the association analysis to variants proved experimentally to be deleterious.

### c. Age-dependent genetic architecture

Inborn errors of type I IFN immunity are more frequent in younger patients (those under the age of 60 years) (61, 74, 76), an observation consistent with IEIs being generally more common in children (26, 83). Eighteen of the 23 patients (78%) first reported to carry biochemically deleterious germline mutations at eight type I IFN-related influenza susceptibility loci were under 60 years old (61). TLR7 deficiency was found to account for 1% of cases of critical COVID-19 in men and about 1.8% of cases of critical COVID-19 in male patients below the age of 60 years (76). In an extended sample of 3269 patients with critical COVID-19, we identified 57 patients carrying a rare predicted LOF variant at 14 type I IFN-related influenza susceptibility loci (including *TYK2*) or with biochemically proven TLR7 deficiency (74). These patients were significantly younger than the rest of the cohort of patients with critical COVID-19 (43.3 vs. 56 years old, *p* = 1.7×10^−5^) (74). Consistent with these results, we recently reported 12 children (~10%) from an international cohort of 112 pediatric patients hospitalized for COVID-19 pneumonia with biochemically complete recessive inborn errors of type I IFN immunity, including seven children with X-linked recessive TLR7 deficiency and five children with autosomal recessive IFNAR1, STAT2, or TYK2 deficiency (67). Interestingly, the effect of the major common genetic risk factor for severe COVID-19 pneumonia on chromosome 3 was also found to be more pronounced in individuals under the age of 60 years than in those over 60 years of age (odds of death or severe respiratory failure of 2.7 vs. 1.5; *P* interaction = 0.038) (84). A greater heritability of common SNPs has also been reported in patients under the age of 60 years than in those over 60 years of age (85).

Stronger genetic effects in young patients may partly reflect the greater contribution of other risk factors in the elderly, such as comorbid conditions and auto-antibodies against type I IFNs (which account for ~15% of critical cases in elderly patients, discussed in a specific section below), which become more frequent with increasing age. At the cellular level, aging is also associated with immunosenescence, which may contribute to a defective innate and adaptive response to SARS-CoV-2 infection, thereby conferring a non-specific predisposition to severe COVID-19 (86). At the molecular level, global type I IFN immunity in the blood (plasmacytoid dendritic cells) and respiratory tract (respiratory epithelial cells) has been shown to decline with age (87–90). The frequency of IEIs may also decline with age in the general population, because IEIs can underlie fatal illness due to influenza or other viruses, resulting in the premature death of affected individuals (2). Cohorts consisting mostly of patients are over the age of 60 years would, therefore, have a very low power to identify rare inborn errors, as illustrated in Figure 2, which shows the proportion of critical COVID-19 cases expected to be due to IEI as a function of age. Assuming a frequency of 10^−3^ for dominant IEIs and 5×10^−4^ for recessive IEIs and a penetrance for critical COVID-19 pneumonia of 0.2 and 0.8, respectively, the expected proportion of critical COVID-19 cases due to IEIs would be expected to decrease strongly with age, from more than 15% below the age of 30 years of age to less than 1% after the age of 60 years.

## 3. Human genetics of susceptibility to SARS-CoV-2 infection

It has been suggested that a fraction of the human population may possess intrinsic resistance to SARS-CoV-2 infection, but this remains unproven (91). Epidemiological observations suggest that some exposed individuals may indeed be resistant; in particular, there have been reports of highly exposed individuals remaining uninfected in the healthcare setting (92), and reports of households in which everyone except one of the spouses became infected (93). Genetic resistance to infection with specific pathogens is rare. The only validated examples in humans are autosomal recessive resistance to: [A] *Plasmodium vivax*, linked to a regulatory variant that modifies the GATA-1 binding site in the *DARC* promoter, thereby selectively preventing the expression of the Duffy antigen on red blood cells (94); [B] HIV-1, conferred by a 32 bp deletion in *CCR5*, the gene encoding the main HIV-1 coreceptor on CD4^+^ T cells (95–97); and [C] norovirus, due to *FUT2* deficiency (non-secretor phenotype), which prevents the binding of the norovirus VPg capsid to FUT2 (98).

No highly penetrant protective variant against SARS-CoV-2 infection has yet been reported. However, GWAS has identified a few genetic factors as associated with a lower likelihood of infection at population level (Figure 1). One of the first genomic regions to be identified in COVID-19 host genetic studies was the ABO locus on chromosome 9 (45). A highly significant association with susceptibility to infection was later observed at the same locus for the SNP rs912805253 (OR ~ 0.9), both in the initial COVID-19 HGI meta-analysis (48) and in a large study by the direct-to-consumer genetics company 23andMe (99). Interestingly, a systematic review confirmed that the ABO association is mostly restricted to susceptibility to infection, with blood group O associated with a significantly lower susceptibility to infection than non-O blood groups (OR = 0.9), whereas most of the other reported genetic regions are associated with disease severity (100). The precise mechanism by which ABO blood status influences SARS-CoV-2 susceptibility remains unclear, but blood antigens are known to alter individual susceptibility to multiple pathogens (101), including other coronaviruses (102).

The angiotensin-converting enzyme 2 (ACE2) protein acts as a functional receptor for the spike glycoprotein of SARS-CoV-2 and other coronaviruses. *ACE2* variants were thus suspected to play a role in modulating infectiousness. However, this gene is under strong negative selection. Putative functional variants are, therefore, rare and a very large number of study participants (>50,000 COVID-19 cases and >700,000 controls) were required to identify a relatively rare variant associated with protection against SARS-CoV-2 infection (rs190509934:T>C, frequency of the minor C allele: 0.3%, OR for the additive effect of each copy of the minor C allele = 0.69) (50). This variant, which is located 69 bp upstream from *ACE2*, is an eQTL for the gene: the C allele is associated with lower levels of mRNA, probably accounting for the lower level of susceptibility to infection. This association was replicated in the most recent HGI meta-analysis (43). Five additional loci were identified by GWAS as being more likely to be associated with susceptibility to SARS-CoV-2 infection than with disease severity (43, 103), but the effect sizes were, again, very modest (Figure 1) and the mechanisms involved remained mostly undefined. No inborn variant conferring strong resistance to SARS-CoV-2 infection has yet been identified. Human genetic studies of resistance to infection may benefit from large-scale host-viral interactome studies (61) and genome-wide CRISPR knockout (104–112) and activation (104, 112) screens, which can identify candidate genes influencing the viral life cycle. Such studies will require a specific strategy, particularly for the reliable identification of highly exposed subjects potentially resistant to infection (113).

## 4. Downstream implications of the genetic findings

### Biological insight from genetic discoveries: auto-Abs neutralizing type I IFNs

The identification of type I IFN-related IEIs led to the almost simultaneous major discovery that pre-existing auto-Abs neutralizing type I IFNs account for about 15% of critical COVID-19 cases (114, 115). While searching for type I IFN-related IEIs in patients with critical COVID-19 pneumonia, we also hypothesized that autoimmune phenocopies of these IEIs might underlie critical COVID-19. Autoimmune phenocopies of IEIs of cytokines have already been described, in which patients with the same, or a similar infectious phenotype produce auto-Abs neutralizing the corresponding cytokines. Auto-Abs against cytokines have already been shown to underlie mycobacterial disease (type II IFN), mucocutaneous candidiasis (IL-17A/F), nocardiosis (GM-CSF), and staphylococcal disease (IL6) (116, 117). Auto-Abs neutralizing type I IFNs were known to occur in some patients receiving IFN therapy, and in patients with systemic lupus erythematosus, myasthenia gravis, thymoma, or autoimmune polyendocrine syndrome (APS-1) caused by germline mutations of *AIRE*, but they were not thought to confer a predisposition to viral diseases (2, 118). We first reported the presence of auto-Abs neutralizing high, supraphysiological concentrations (10 ng/mL, with plasma diluted 1/10) of IFN α2 and/or IFN ω in about 10% of 987 patients with critical COVID-19 pneumonia, but not in 663 individuals with asymptomatic or mild infection (115). This finding has been largely replicated worldwide in many studies (76, 119–134). We later detected auto-Abs neutralizing lower, more physiological concentrations (100 pg/mL, with plasma diluted 1/10) of IFN α2 and/or IFN ω in 13.6% of 3595 patients with life-threatening COVID 19 (114). This proportion increased in patients older than 65 years, reaching more than 20% in patients over 80 years old, and was greater in men than in women. Another 1% of patients with critical COVID-19 had auto-Abs neutralizing high concentrations of IFN-β.

Several lines of evidence strongly suggest that autoimmunity to type I IFN plays a causal role in life-threatening COVID-19 (135). For patients for whom plasma sampled before the pandemic was available, the auto-Abs were found to be present before SARS-CoV-2 infection (115). Patients with autoimmune polyendocrine syndrome type-1 (APS-1), who produce such auto-Abs from early childhood, were shown to be at very high risk of developing severe or critical COVID-19 pneumonia, especially after the age of 20 years (136). These auto-Abs neutralize the antiviral activity of type I IFNs against SARS-CoV-2 *in vitro* (115) and are found *in vivo* in the blood and in the respiratory tract of patients (123, 137, 138). Remarkably, these auto-Abs were also found in samples from a fraction of the general population before the pandemic. Their prevalence in the general population remains fairly stable until the age of 70 years (at ~1% for auto-Abs neutralizing low doses of IFN α2 and/or IFN ω), but sharply increases thereafter (reaching up to 6.3% after the age of 80 years) (114). Auto-Abs against type I IFNs strongly increase COVID-19 infection fatality rates (IFRs) in unvaccinated populations, especially those neutralizing both IFN-α2 and –ω (135). Screening for auto-Abs against type I IFN in patients infected with SARS-CoV-2, and even in uninfected individuals, is feasible and may be warranted. Individuals carrying such antibodies should be given high priority for vaccination against COVID-19. They may also benefit from specific care, such as the administration of monoclonal antibodies neutralizing the virus or early recombinant IFN-β therapy (139). The exact level of protection against severe COVID-19 pneumonia provided by COVID-19 vaccines in carriers of auto-Abs remains unclear. However, we recently detected auto-Abs against type I IFNs in 24% (10 of 48 tested) of fully vaccinated patients with normal antibody responses who developed critical breakthrough COVID-19 (140), suggesting that at least some of the carriers of these auto-Abs may not be fully protected by the vaccine. The same auto-Abs were subsequently shown to underlie other severe infectious manifestations, such as severe adverse reactions to yellow fever live attenuated viral vaccine (33) and critical influenza pneumonia (141). In critically ill COVID-19 patients, auto-Abs were also shown to increase the risk of herpesvirus reactivation, which has been associated with a poorer clinical outcome (142, 143).

Auto-Abs against type I IFNs can also be genetically driven and few IEIs are already known to underlie their production. The most striking example is the production of these auto-Abs from early childhood in nearly all patients with APS-1 due to germline deleterious variants of *AIRE* (4). They have also been reported in patients with immunodysregulation polyendocrinopathy enteropathy X-linked (IPEX) due to deleterious variants of *FOXP3*, and combined immunodeficiency due to biallelic hypomorphic *RAG1* or *RAG2* variants (37–42). A feature common to these IEIs is that they affect T-cell tolerance. Interestingly, among the patients with auto-Abs against type I IFN and life-threatening COVID-19, we identified a woman with X-linked incontinentia pigmenti (IP), in which cells activate the same single X chromosome (cells having activated the X chromosome bearing the null mutation of *NEMO* dying during development) (27). A further study of 32 women with IP showed that 25% carried auto-Abs against type I IFNs, suggesting an X-linked germline genetic etiology for type I IFN auto-Abs (115). New IEIs underlying the production of auto-Abs against type I IFN are likely to be discovered in the future, particularly in young patients. It is also tempting to speculate that somatic mutations, which accumulate with aging in normal human tissues (144), may be partly responsible for the sharp increase in the prevalence of auto-Abs against type I IFNs after the age of 70 years.

### Clinical implications: towards personalized medicine in infectious diseases

Deciphering the genetic architecture of susceptibility to SARS-CoV-2 infection and severe COVID-19 pneumonia is only the first step toward clinical implementation and improved healthcare. How can we translate the knowledge gained in the genomic screens described above into medically useful strategies? The first and most obvious answer to this question is through the identification of individuals at high risk. The rare deleterious variants of *TLR7* and TLR3-dependent type I IFN immunity genes identified confer a massive increase in the risk of severe disease and account for a significant proportion of cases (e.g. 1% of men with critical disease carry a deleterious *TLR7* variant). One could imagine screening programs, at population level, primary care centers or emergency rooms (at the time of early COVID-19 diagnosis), providing point-of-care testing for such variants (145). Carriers would then benefit from specific preventive and therapeutic measures. In particular, type I IFN might be useful in patients prone to severe COVID-19 and infected with the virus, provided it is administered at an early stage of infection. Along the same lines, the discovery that auto-antibodies against type I IFN account for 20% of COVID-19 deaths have clinical implications. The detection of such auto-Abs before or during early stages of infection is straightforward and warranted. Carriers of these auto-Abs should be vaccinated and given priority for booster injections and may benefit from specific treatments, such as IFN-β, mAbs neutralizing SARS-CoV-2, or plasma exchange (146, 147).

A second way forward in the clinical translation of these findings would be the development of polygenic risk scores (PRS), constructed by summing the effects of all genetic variants confirmed to be associated with a phenotype of interest. In recent years, PRS have been shown to have a high predictive value for a range of complex diseases, including cardiovascular, metabolic, and tumoral disorders (148). A few studies have attempted to build PRS for COVID-19, but with little success. Indeed, one of the main factors determining the predictive ability of PRS is the fraction of the phenotypic variance explained by the combination of selected variants, which remains low in COVID-19 host genetic studies of common variants (50, 84, 149). Nevertheless, some private companies already offer polygenic risk prediction for severe COVID-19. However, current tests have low discriminatory power at the individual level and variable accuracy depending on ancestry, making their clinical use questionable (150). Nevertheless, the assessment of patient risk based on a combination of demographic, clinical and genetic data has the potential to deliver more precise information that could prove useful for personalized health management.

## 5. Conclusion

The SARS-CoV-2 pandemic has highlighted the vast potential of human genomics research when it is performed at a large scale, in real time, and in a highly collaborative manner. One of its greatest successes has been the identification of a molecular explanation for about 20% of cases of critical COVID-19 pneumonia: inborn errors of type I IFN immunity in 1–5% of cases and auto-Abs against type I IFNs in 15–20% of cases. Other IEIs, related or unrelated to type I IFN, may also be involved. Future studies should build on the observations of the impact of SARS-CoV2 infection in individuals with previously known IEIs (151). Remarkably, common genetic variants with modest effect sizes were also identified in regions encompassing genes involved in type I IFN immunity. It is tempting to speculate that they may act as modifiers of the clinical expression of IEIs, which display high but incomplete penetrance. Most human genomics research studies have focused on COVID-19 pneumonia, with fewer considering resistance to SARS-CoV-2 infection. Interesting results are starting to emerge for MIS-C and already suggest a pathogenesis different from that of COVID-19 pneumonia (152–154). Future research studies should also encompass other COVID-19-related clinical manifestations, such as long-COVID (13) and COVID-toes (12), as well as severe adverse effects of vaccination (155) and breakthrough infections (140).

The lessons learned should be used to improve our collective capacity to confront other infectious threats. In particular, COVID-19 has illustrated the central importance of large-scale research infrastructures embedded in healthcare systems, facilitating the rapid collection and analysis of samples in times of crisis. It has also become clear that it is crucial to define disease outcomes clearly and to gather as much clinical data as possible to minimize patient misclassification, thereby maximizing statistical power to detect true genetic signals. Ultimately, a better understanding of the impact of human genetic variation on pathogen response will enable health systems to provide appropriate care to protect individuals and populations more efficiently against future infectious threats.
